# Ablation of NLRP3 inflammasome attenuates muscle atrophy via inhibiting pyroptosis, proteolysis and apoptosis following denervation

**DOI:** 10.7150/thno.74831

**Published:** 2023-01-01

**Authors:** Zongqi You, Xinying Huang, Yaoxian Xiang, Junxi Dai, Lei Xu, Junjian Jiang, Jianguang Xu

**Affiliations:** 1 Department of Hand Surgery, Huashan Hospital, Fudan University, Shanghai, China.; 2 Key Laboratory of Hand Reconstruction, Ministry of Health, Shanghai, China.; 3 Shanghai Key Laboratory of Peripheral Nerve and Microsurgery, Shanghai, China.; 4 School of Rehabilitation Science, Shanghai University of Traditional Chinese Medicine, Shanghai, China.

**Keywords:** NLRP3 inflammasome, denervation, muscle atrophy, pyroptosis, proteolysis, apoptosis

## Abstract

**Rationale:** The inflammasome has been widely reported to be involved in various myopathies, but little is known about its role in denervated muscle. Here, we explored the role of NLRP3 inflammasome activation in experimental models of denervation *in vitro* and *in vivo*.

**Methods:** Employing muscular NLRP3 specific knock-out (NLRP3 cKO) mice, we evaluated the effects of the NLRP3 inflammasome on muscle atrophy *in vivo* in muscle-specific NLRP3 conditional knockout (cKO) mice subjected to sciatic nerve transection and *in vitro* in cells incubated with NLRP3 inflammasome activator (NIA). To evaluate the underlying mechanisms, samples were collected at different time points for RNA-sequencing (RNA-seq), and the interacting molecules were comprehensively analysed.

**Results***:* In the experimental model, NLRP3 inflammasome activation after denervation led to pyroptosis and upregulation of MuRF1 and Atrogin-1 expression, facilitating ubiquitin-proteasome system (UPS) activation, which was responsible for muscle proteolysis. Conversely, genetic knockout of NLRP3 in muscle inhibited pyroptosis-associated protein expression and significantly ameliorated muscle atrophy. Furthermore, cotreatment with shRNA-NLRP3 markedly attenuated NIA-induced C2C12 myotube pyroptosis and atrophy. Intriguingly, inhibition of NLRP3 inflammasome activation significantly suppressed apoptosis.

**Conclusions:** These *in vivo* and *in vitro* findings demonstrate that during denervation, the NLRP3 inflammasome is activated and stimulates muscle atrophy via pyroptosis, proteolysis and apoptosis, suggesting that it may contribute to the pathogenesis of neuromuscular diseases.

## Introduction

Peripheral nerve injury caused by trauma accounts for approximately 2% of all limb injuries worldwide 1. Such injuries result in a rapid and progressive reduction in muscle size and muscle atrophy 2. Although advancements in microsurgical techniques have been made for decades, one-third of patients with peripheral nerve injury achieve poor functional recovery 3. Previous studies have suggested that poor functional recovery is caused by disruption of protein homeostasis, which results in reduced protein synthesis and enhanced protein degradation through the ubiquitin-proteasome system (UPS); however, the underlying mechanism remains to be determined 4. Recently, accumulating studies have demonstrated that inflammatory signalling pathways play crucial roles in inducing muscle atrophy 5-7. However, the exact pathophysiological mechanisms underlying this process are currently unknown.

Pyroptosis, a highly inflammatory from of programmed cell death that is distinct from apoptosis and necrosis, is initiated by the activation of the NOD-like receptor protein 3 (NLRP3) inflammasome 8. Afterwards, the cytokine precursors pro-IL-1β and pro-IL-18 are converted into active cytokines after pro-caspase 1 is cleaved by the NLRP3 inflammasome 9. Additionally, caspase 1 can generate an N-terminal fragment of gasdermin D (GSDMD-N), which induces pyroptosis by forming plasma membrane pores 10. Pyroptotic cell death occurs immediately following gasdermin D (GSDMD) pore formation, and a significant amount of IL-1β is released from GSDMD pores 11. Although several groups recently reported that the inflammasome plays a key role in muscular diseases 12, 13, the link between the NLRP3 inflammasome and muscle atrophy induced by denervation is still unclear.

Given that the UPS seems closely tied to muscle atrophy, it is tempting to speculate that NLRP3 inflammasome-induced pyroptosis contributes to UPS activation during denervation to accelerate muscle atrophy. The efficient release of mature IL-1β mainly results from NLRP3 inflammasome activation 14. IL-1β is considered a critical host-protective cytokine during infection; however, due to the “sterile” settings, it plays various roles during denervation. Emerging evidence reveals that IL-1β increases the levels of the E3 ligase muscle RING finger (MuRF) 1 and F-box protein atrogin 1 expression, which are reliable markers of protein catabolism during atrophic conditions, including denervation 15, in muscle 16, 17.

As there are few proven strategies for alleviating muscle atrophy following denervation, novel therapeutic targets are needed. We provide evidence for the importance of the NLRP3 inflammasome, pyroptosis and cytokines such as IL-1β in denervation in the present study. Here, we employ an *in vivo* mouse model of denervated muscle atrophy and an *in vitro* cellular model of denervation induced by NLRP3 inflammasome activator (NIA). Overall, our findings reveal the role of the NLRP3 inflammasome in the denervation-induced muscle atrophy and the underlying mechanism.

## Materials and methods

### Animals

C57BL/6J male mice and NLRP3 conditional knockout (cKO) mice were obtained from Shanghai Model Organisms Center, Inc. (Shanghai, China) and used in this study. NLRP3 cKO mice were generated as described below. A NLRP3-targeting vector was constructed by ET cloning techniques in EL250 bacterial cells. The targeting vector was designed to flank exons 2-3 with loxp sites and a pGK- polyA cassette. ES cells from C57BL/6J mice were electroporated with the targeting vector. Then, the homologous recombination clones were subjected to double drug selection with G418 and ganciclovir. Through long PCR and sequencing, the ES clones with high resistance were identified. To generate chimeric offspring, ES cell clones were expanded and injected into blastocysts of C57BL/6J mice. Then, heterozygous floxed NLRP3 (NLRP3^flox/+^) mice were obtained by crossing the chimeric mice with C57BL/6J mice. NLRP3^flox/+^ mice were crossed with Myf5-Cre mice to generate Myf5^Cre/+^/NLRP3^flox/flox^ (NLRP3 cKO) and Myf5^+/+^/NLRP3^flox/flox^ wild-type (WT) mice. Throughout the experiment, the mice housed at a constant temperature of 24 °C and humidity of approximately 45% on a 12 h light/dark cycle.

### C2C12 myoblast culture, differentiation and transfection

Murine C2C12 myoblasts were obtained from American Type Culture Collection (ATCC) and incubated in DMEM supplemented with 80 U/ml penicillin, 0.08 mg/ml streptomycin and 10% foetal bovine serum (Gibco, CA, United States) at 37 °C, 5% CO_2_. Isolated subconfluent myoblasts were cultured in DMEM containing 2% horse serum (Biological Industries, Israel) for 4 d to allow the formation of mature myotubes. A lentivirus (LV) expressing shRNA-GFP-NLRP3 (sh-NLRP3) was obtained from Zorin Biotech (Shanghai, China). The sequence of sh-NLRP3 was GCAGGTTCTACTCTATCAAGGCCTTGATAGAGTAGAACCTGC. Then, normal C2C12 myotubes were transfected with LV expressing sh-NLRP3 for 48 h, after which fluorescence was observed. Finally, puromycin was employed for screening, and stably transfected cells were selected.

### Animal model

Male C57BL/6J mice, WT (C57BL/6J genetic background, male) mice and NLRP3 cKO (C57BL/6J genetic background, male) mice aged 6-8 weeks and weighing 22-25 g were employed in this study. The mice underwent surgical denervation of the gastrocnemius (GAS) as described by Dai et al 18. Before surgery, the mice were anaesthetized with 0.2% isoflurane, and 0.1 mg/kg buprenorphine (analgesic) was administered. The GAS muscles were denervated by removing a small section of the sciatic nerve of the left hind leg. Then, 4-0 absorbable sutures were used to close the skin. While the mice recovered from anaesthesia, they were closely observed until their wounds were sutured. After denervation, the mice were sacrificed at various time points (0, 1, 3, 5, 7, 14, 21, 28 d) by cervical dislocation, with 6 mice being sacrificed at each time point. Sham mice were subjected to the same procedures except sciatic nerve transection. At each time point, the GAS muscles were removed and weighed and then stored at -80 °C until further analysis. The experimental protocols performed in this study were approved by Fudan University's Animal Care and Use Committee and conformed to the guidelines of the National Institute of Health Guide for the Care and Use of Laboratory Animals.

### Cell model

To induce NLRP3 activation, 100 ng/mL NIA (Abcam, Cambridge, United Kingdom) was added to the culture medium of C2C12 myotubes. According to the results of a preliminary experiment, the C2C12 myotubes were stimulated with NIA for approximately 12 h, 1 d or 3 d to mimic denervation.

### Force and wet weight measurement

At various time points, the mice were firstly anaesthetized. Before sacrifice, the muscle function was evaluated *in situ*. The end of GAS tendon was isolated and attached to a force transducer (KaiHuiSD Technology, Beijing, China). Next, the muscle length was adjusted to provide maximal tetanic force and the muscle were stimulated with fixed frequency. The force was recorded as newtons per square centimeter (N/cm^2^). The force loss was defined as the post-denervation production compared to the nondenervated side. After that, the mice were sacrificed by cervical dislocation and the left and right GAS muscles were removed, washed with saline and weighed. The wet weight ratio was calculated as the wet weight of the muscle on the uninjured side divided by the wet weight of the muscle on the side of nerve injury. After that, the GAS muscles were placed in 4% paraformaldehyde and stored at -80 °C until use.

### Haematoxylin-eosin (HE) staining

Fresh GAS muscle samples were fixed with 4% paraformaldehyde overnight, embedded in paraffin and subjected to HE staining. Images were captured using a microscope (Zeiss Axio Scope.A1, Germany), and the area of each fibre cross-section in six randomly chosen fields was calculated with ImageJ software (National Institutes of Health, USA).

### RNA sequencing (RNA-seq)

Following the manufacturer's instructions, total RNA was extracted from muscle tissue samples using the miRNA Isolation Kit (Thermo Fisher Scientific, Waltham, MA, USA). The purity of each sample was determined by a NanoPhotometer ® (IMPLEN, CA, USA). Then, an Agilent 2100 RNA Nano 6000 Kit (Agilent Technologies, CA, USA) was used to determine the concentration and integrity of the RNA samples. A total of 1-3 μg of RNA per sample was used as the input material for RNA sample preparation. According to the manufacturer's recommendations, RNA-seq libraries were generated with the VAHTS Universal V6 RNA-seq Library Prep Kit for Illumina ® (NR604-01/02), and each sample was assigned index codes based on its attributed sequences. After library examination, index-coded samples were clustered using the HiSeq PE Cluster Kit v4-cBot-HS (Illumina) on a cBot cluster generation system. Finally, 150-bp paired-end reads were generated on the Illumina platform for each library.

### Western blot analysis

First, equal amounts of protein were leaded in each lane of a polyacrylamide gel. Next, the proteins were separated by SDS-PAGE and transferred to a PVDF membrane. After incubated for two hours at room temperature, the membrane was blocked with 5% skim milk and sequentially incubated with primary antibodies against NLRP3 (Cell Signaling Technology, Danvers, MA, USA), GSDMD (Biorbyt, St Louis, MO, USA), ASC (Biorbyt), Caspase 1/p20/p10 (Proteintech, Rosemont, USA), MuRF1 (Biorbyt), Atrogin-1 (Biorbyt), Caspase 3 (Proteintech), IL-1β (Biorbyt), IL-18 (Sigma-Aldrich, St. Louis, MO, USA) and glyceraldehyde 3-phosphate dehydrogenase (GAPDH) (Bioworld Technology, Minneapolis, MN, USA) overnight at 4 °C. The membrane was then washed and incubated for 2 h at room temperature with horseradish peroxidase-conjugated IgG. Finally, we employed ECL Western blotting detection reagents (Thermo Fisher Scientific) to visualize the protein bands and used Un-Scan-It 6.1 software (Silk Scientific, Orem, UT, USA) to measure the band density.

### Isolation of total RNA and real-time PCR

RNA was isolated from C2C12 myotubes and GAS muscle tissues using and RNA Extraction Kit (Sigma-Aldrich). The PrimeScript™ RT Reagent Kit (Perfect Real Time, Takara, Japan) was employed to synthesize cDNA. Quantitative real-time PCR was performed on a QuantStudio 5 system (Applied Biosystems, USA) using Maxima SYBR Green/ROX qPCR Master Mix (2X) (Thermo Fisher Scientific). GAPDH was utilized as an internal control to determine the relative gene expression levels. All primer sequences are listed in Table 1.

### Immunofluorescence staining

Briefly, GAS muscle sections (6 μm thickness) or C2C12 myotubes were incubated with primary antibody against NLRP3 (Cell Signaling Technology) overnight at 4 °C. The sections were washed three times with PBS and incubated with secondary antibodies (Alexa Fluor^TM^ 594-conjugated, Jackson ImmunoResearch Laboratories, West Grove, PA, USA) for 1 h at room temperature and then counterstained with myosin (Alexa Fluor^TM^ 488-conjugated, Thermo Fisher Scientific) overnight at 4 °C. At last, they were incubated with 4,6-diamidino-2-phenylindole (DAPI, Millipore Sigma, Burlington, MA, USA) for 4 min at room temperature. Finally, a Zeiss HB050 inverted microscope (Carl Zeiss, Oberkochen, Germany) was used to examine the fluorescence.

### Enzyme-linked immunosorbent assay (ELISA)

GAS muscle tissues and the medium of C2C12 myotubes were collected and centrifuged for 20 min at 4 °C and 12,000 rpm. We measured the levels of IL-1β and IL-18 using ELISA kits (R&D Systems, Minneapolis, MN, USA) according to the manufacturer's instructions.

### TUNEL staining

As described in previous studies, a TUNEL Detection Kit (Roche, Indianapolis, IN, USA) was employed for TUNEL staining. In detail, after overnight incubation with primary antibody at 4 °C, GAS muscle sections (6 μm thickness) or C2C12 myotubes were incubated with TUNEL reaction mixture for 1 h at 37 °C in the dark. They were then stained with DAPI (Sigma-Aldrich) for 2 min after 3 washes with PBS.

### Flow cytometry

After 3 washes with PBS, GAS muscle tissues or C2C12 myotubes were resuspended in fluorescein isothiocyanate (FITC)-conjugated Annexin-V binding buffer. Then, 5 µl FITC-conjugated Annexin-V (Keygen Biotech, Jiangsu, China) was added, and the samples were incubated in a dark chamber. The reaction mixture was kept at room temperature for 15 min. In the end, the samples were stained with 5 µl of propidium iodide (PI), incubated for 10 min, and analysed by flow cytometry (Becton-Dickinson FACSCalibur, United States).

### Statistical analysis

The data in the present study are presented as the means ± SDs. Differences between the two experimental groups were analysed by unpaired 2-tailed Student's t tests. Differences among multiple groups were analysed by 1- or 2- way ANOVA followed by Bonferroni's post hoc test. Significance was set at *p* < 0.05.

## Results

### Morphological and structural changes after experimental denervation

**Figure 1:**
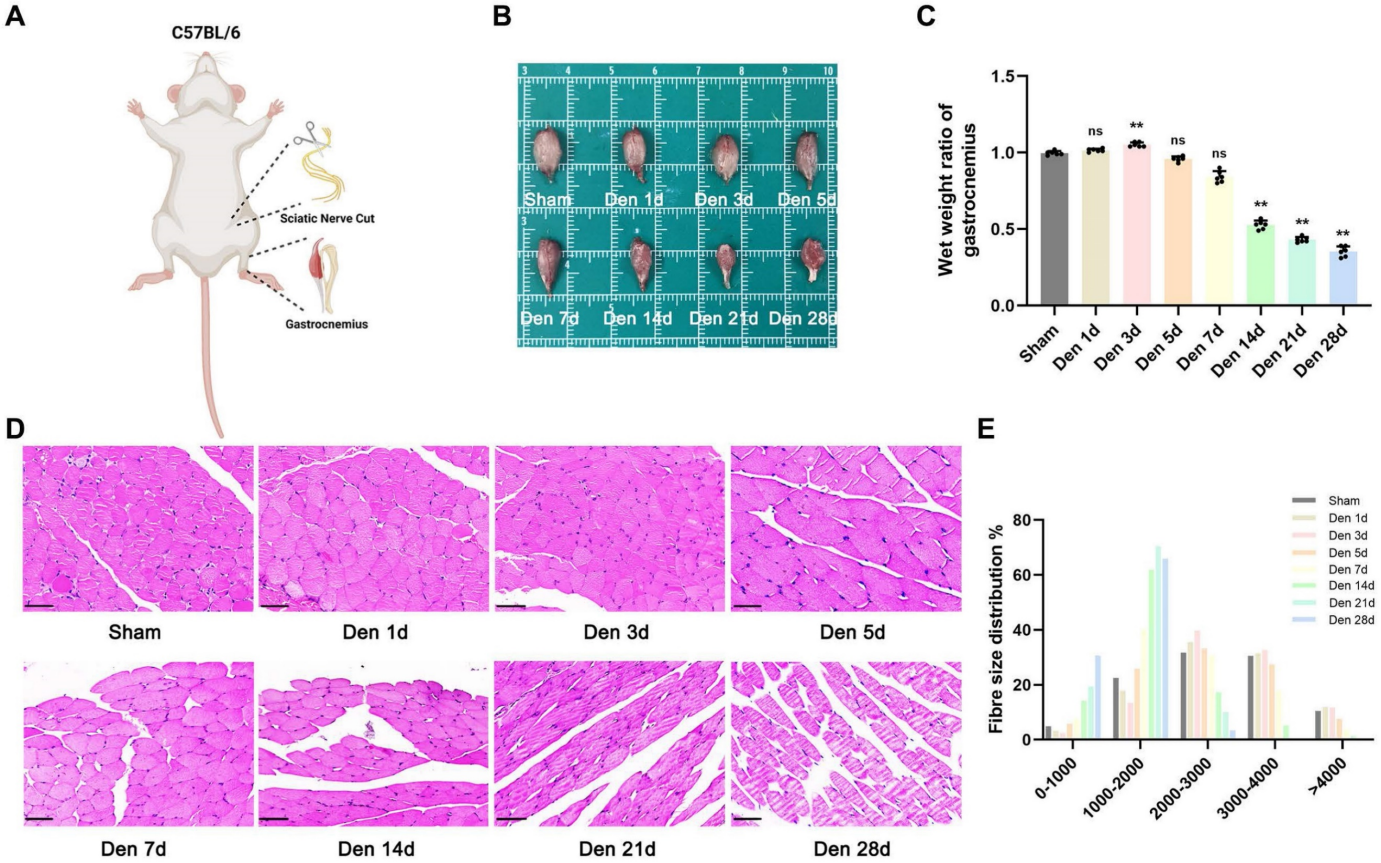
** Denervation led to atrophy of GAS. (A)** The abridged schematic diagram of sciatic nerve transection model in mice. **(B)** General observation of the GAS muscles at indicated time points after denervation. **(C)** The wet weight ratio (operational side weight / contralateral side weight) and average fibre diameter of GAS at indicated time points after denervation. **(D)** Morphological observation of GAS muscles by HE staining at indicated time points after denervation. Scale bar, 50 μm. **(E)** Fibre size distribution of GAS muscles at indicated time points after denervation. Data are expressed as mean ± SD. n = 6 per group. *p < 0.05, **p < 0.01, ns *p* > 0.05 vs Sham group. Den, denervation. GAS, gastrocnemius.

We established a sciatic nerve transection mouse model and explored the progression of GAS muscle atrophy after denervation (Figure 1A). As previously reported 19, the wet weight ratio of GAS muscle showed an upwards trend during the first 3 d after injury, possibly due to short-term oedema, as indicated rapid weight loss (approximately 67%) over the next 25 d (Figure 1B-D). Notably, no significant difference in the wet weight ratio was seen within 5 d after denervation (Figure 1C). Furthermore, the morphology of muscle fibres was not different between the model group and sham group until the 14^th^ d after denervation (Figure 1C). In general, analysis of GAS muscle tissues with timeline manifestation provided intuitive evidence of the morphological and structural changes that occurred after experimental denervation and indicated that extensive atrophy may occur beginning 14 d after injury.

### Analysis of differentially expressed genes (DEGs) and Gene Ontology (GO) biological process (BP) enrichment after experimental denervation

**Figure 2:**
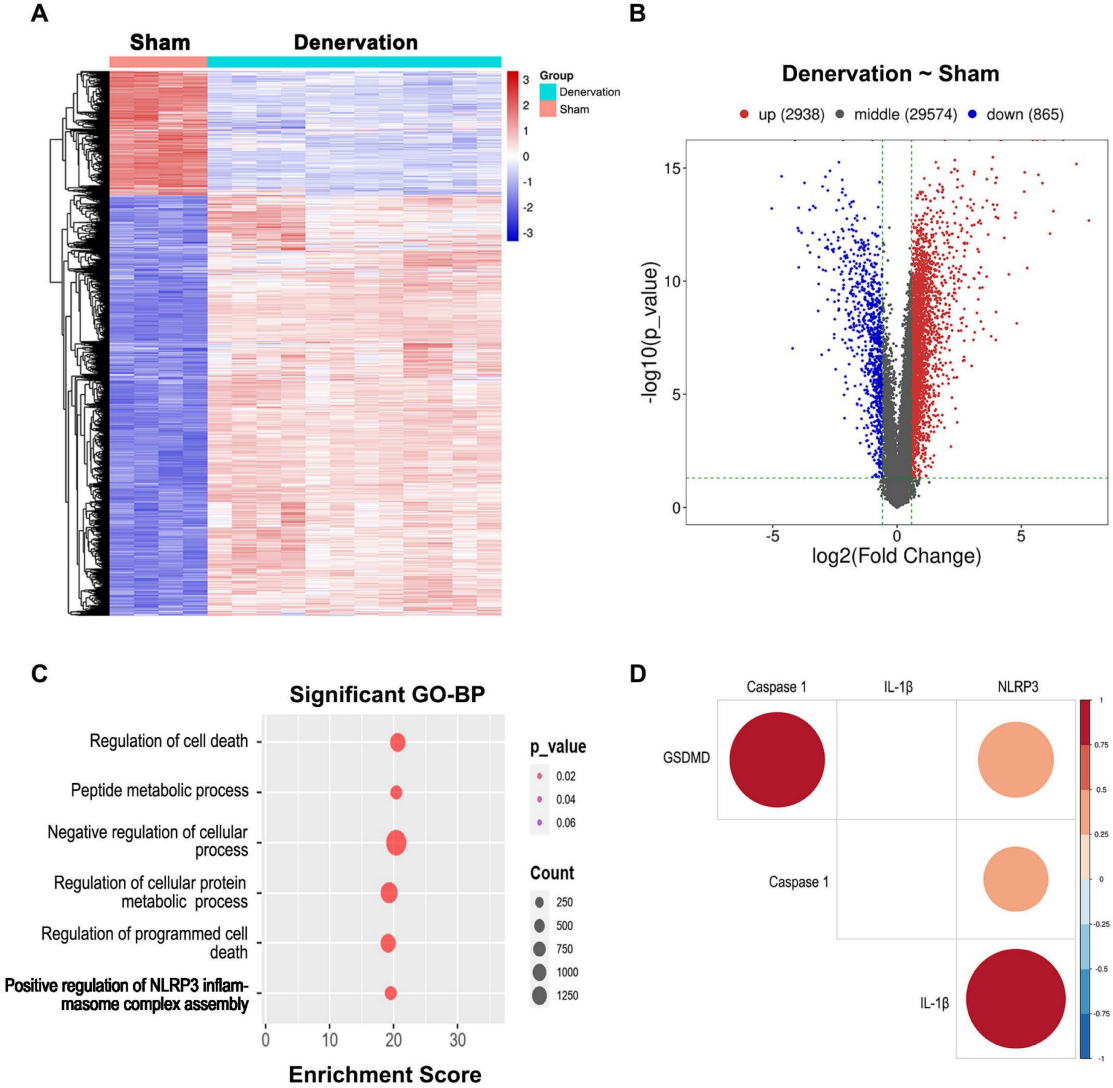
** Deep changes in GAS transcriptome after denervation. (A)** Heatmap from GAS muscle transcripts. **(B)** Volcano plot from GAS transcripts, red indicates high and blue indicates low expression level of the 33377 genes. **(C)** BP distribution based on GO analysis from GAS transcripts. **(D)** Correlation analysis between NLRP3 and pyroptotic molecules based on GAS transcripts. BP, biological process. GO, gene ontology.

RNA extracted from GAS muscle tissues, and DEG and GO-BP term enrichment analyses were performed. To ensure the reproducibility of the results, at least four biologically independent samples were used in each analysis. A total of 33377 DEGs, including 865 downregulated and 2938 upregulated genes were identified between atrophied GAS muscle tissues and GAS muscle tissues from the sham (nondenervated) group at 14, 21 and 28 after denervation; these DEGs are presented in a heatmap (Figure 2A) and a volcano plot (Figure 2B). Moreover, GO enrichment analysis of the DEGs was performed, and the BP terms in which the upregulated genes were enriched are shown in Figure 2C.

Notably, genes that showed upregulated expression during atrophy were enriched in “positive regulation of NLRP3 inflammasome complex assembly” (Figure 2C), suggesting that the NLRP3 inflammasome might be activated during this process. Due to the crucial role of the NLRP3 inflammasome in initiating pyroptosis 10, we further investigated the correlation between the NLRP3 level and the levels of pyroptosis-related genes based on the RNA-seq data. Significant correlations between the NLRP3 level and the levels of pyroptosis-related molecules, containing Caspase 1, GSDMD and IL-1β (Figure 2D), were observed, indicating that NLRP3-mediated pyroptosis participates in atrophy after denervation.

### NLRP3 inflammasome-mediated pyroptosis in denervated GAS muscles

**Figure 3:**
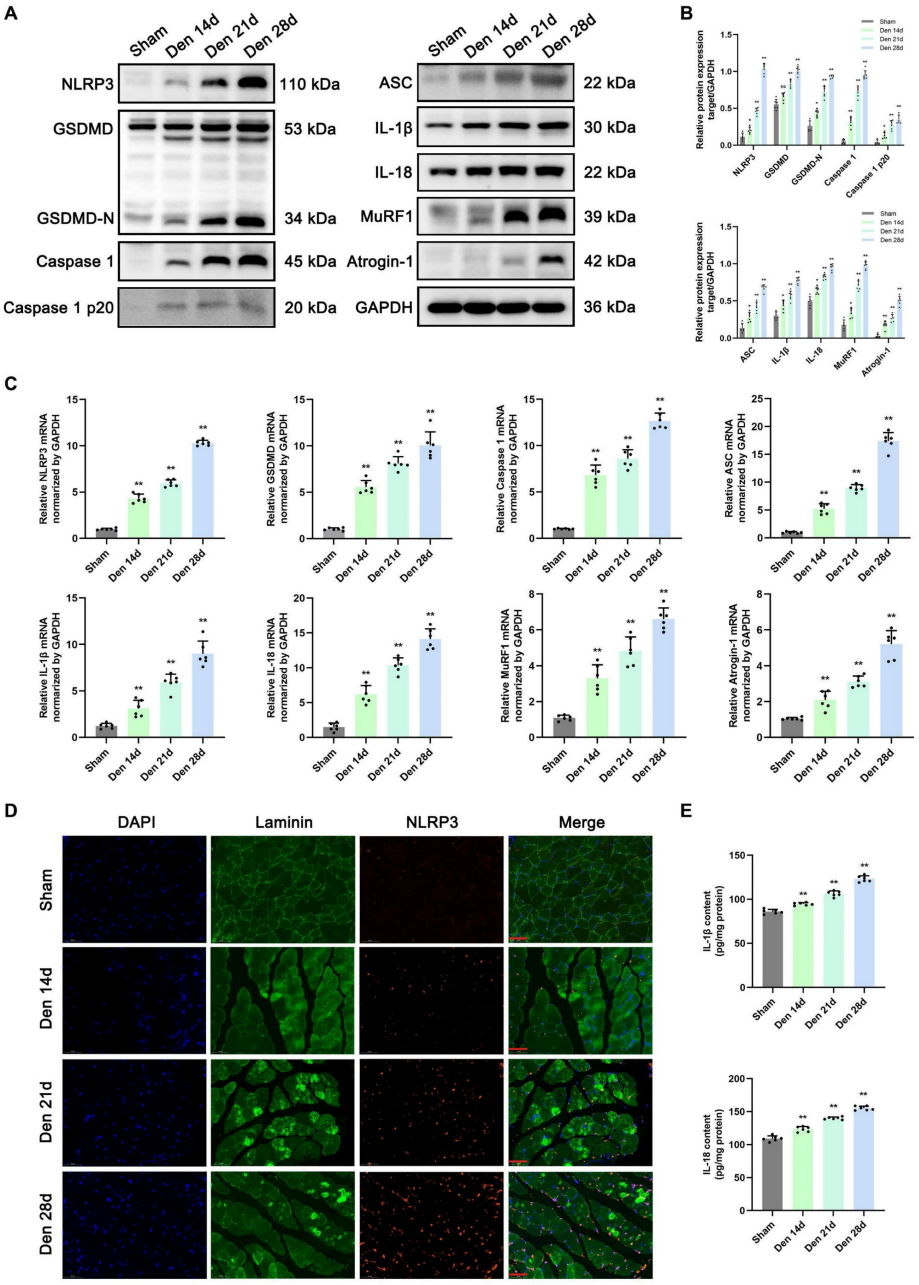
** Denervation induces NLRP3 inflammasome activation accompanied by pyroptotic molecules and UPS ligases upregulation in atrophic GAS. (A)** Western blot analysis of expression levels of NLRP3, GSDMD, GSDMD-N, Caspase 1, Caspase 1 p20, ASC, IL-1β, IL-18, MuRF1 and Atrogin-1 at 0, 14, 21 and 28 d after denervation. **(B)** Quantification of NLRP3, GSDMD, GSDMD-N, Caspase 1, Caspase 1 p20, ASC, IL-1β, IL-18, MuRF1 and Atrogin-1. **(C)** Quantitative PCR analysis of mRNA levels of NLRP3, GSDMD, Caspase 1, ASC, IL-1β, IL-18, MuRF1 and Atrogin-1 at 0, 14, 21 and 28 d after denervation.** (D)** Representative immunofluorescence staining images of NLRP3 at 0, 14, 21 and 28 d after denervation. Scale bar, 50 μm. **(E)** Quantitative ELISA analysis of IL-1β and IL-18 in GAS at 0, 14, 21 and 28 d after denervation. Data are expressed as mean ± SD. n = 6 per group. *p < 0.05, **p < 0.01, ns *p* > 0.05 vs Sham group. Den, denervation. GAS, gastrocnemius.

To clarify the role of NLRP3 inflammasome-mediated pyroptosis in muscle atrophy, the expression of pyroptosis-related molecules and atrophy markers in GAS muscle was measured post denervation. Western blotting and PCR were performed to assess the levels of components of pyroptotic and atrophic pathways, including NLRP3, GSDMD, GSDMD-N, caspase 1, caspase1 p20, ASC, IL-1β, IL-18, MuRF1 and Atrogin-1, at different time points after denervation. The mRNA and protein levels of pyroptosis-related molecules were significantly increased at 14 d after denervation and showed a continued increasing trend of up-regulation over the next 14 d (Figure 3A-C). Moreover, the expression levels of MuRF1 and Atrogin-1 gradually increased in denervated GAS muscles, which is consistent with the changes of pyroptosis-related signals (Figure 3A-C).

Then, double immunofluorescence staining was performed to assess the localization of the NLRP3 inflammasome in denervated GAS muscles. As shown in Figure 3D, in the sham group, NLRP3 was weakly expressed in the muscles. However, the expression levels of NLRP3 were increased at 14 d post denervation and increased over time. In contrast, myosin expression significantly decreased as muscle atrophy progressed (Figure 3D). ELISA, Western blotting, and PCR revealed that IL-1β and IL-18 levels were markedly increased in the model group compared with the sham group from 14 to 28 d after denervation (Figure 3E). The above results indicated that the mRNA and protein levels of NLRP3 inflammasome-related molecules are markedly increased at 28 d after denervation, and these changes may contribute to UPS activation and muscle atrophy.

### NLRP3 cKO in skeletal muscles attenuated the effects of GAS muscle denervation

**Figure 4:**
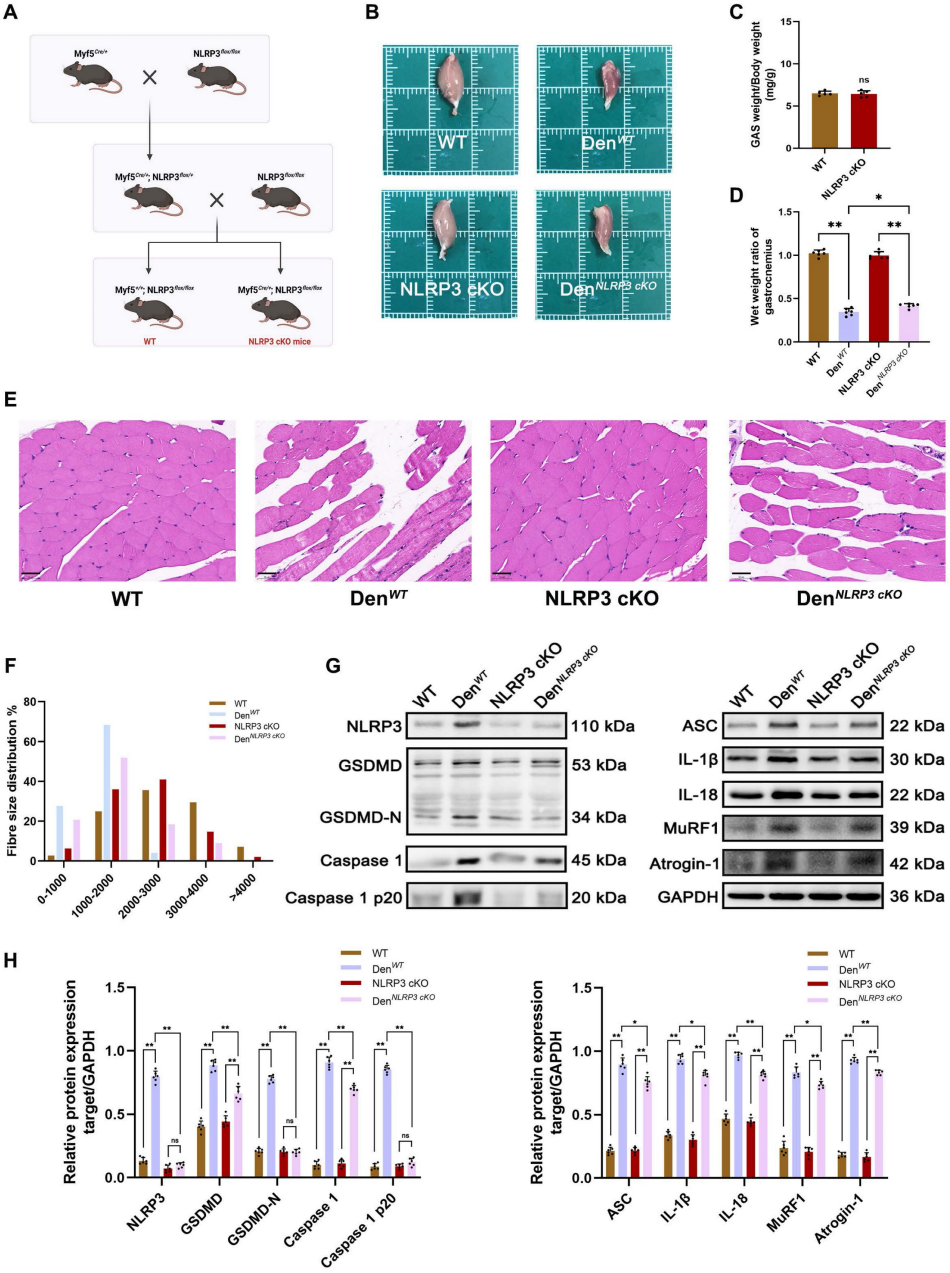
** NLRP3 cKO attenuates muscle atrophy and pyroptosis after denervation. (A)** Outline of the scheme to obtain WT and NLRP3 cKO mice. **(B)** General observation of the GAS in each group.** (C)** Quantification of GAS muscles weight / body weight in WT and NLRP3 cKO mice in each group. **(D)** The wet weight ratio (operational side weight / contralateral side weight) of GAS muscles in each group. **(E)** Morphological observation of GAS by HE staining in each group. Scale bar, 50 μm. **(F)** Fibre size distribution of GAS muscles in each group. **(G)** Western blot analysis of expression levels of NLRP3, GSDMD, GSDMD-N, Caspase 1, Caspase 1 p20, ASC, IL-1β, IL-18, MuRF1 and Atrogin-1 in each group. **(H)** Quantification of NLRP3, GSDMD, GSDMD-N, Caspase 1, Caspase 1 p20, ASC, IL-1β, IL-18, MuRF1 and Atrogin-1. Data are expressed as mean ± SD. n = 6 per group. *p < 0.05, **p < 0.01, ns *p* > 0.05. WT, wild type. Den, denervation.

To explore the main roles of the NLRP3 inflammasome in muscle atrophy after denervation, NLRP3 cKO mice were employed. In brief, mice carrying floxed NLRP3 alleles with loxP sites were crossed with mice expressing Cre recombinase at the Myf5 locus (Myf5^Cre/+^) to generate Myf5^Cre/+^/NLRP3^flox/flox^ (NLRP3 cKO) mice and control (Myf5^Cre/+^/NLRP3^flox/flox^) littermates (referred to as WT mice) (Figure 4A). Given that NLRP3 expression was persistently upregulated in denervated GAS muscles and peaked at 28 d after denervation, GAS muscle samples were collected at this time point. Notably, the volume and weight of GAS muscles from NLRP3 cKO mice were not significantly different from those of GAS muscles from WT mice (Figure 4B-C); however, an obvious increase in the GAS muscle wet weight ratio was observed in NLRP3 cKO mice compared with WT mice after denervation (Figure 4D). Consistently, HE staining revealed that atrophy of the denervated GAS muscle was significantly attenuated in NLRP3 cKO mice (Figure 4E), and the average fibre diameter showed a similar change (Figure 4F).

**Figure 5:**
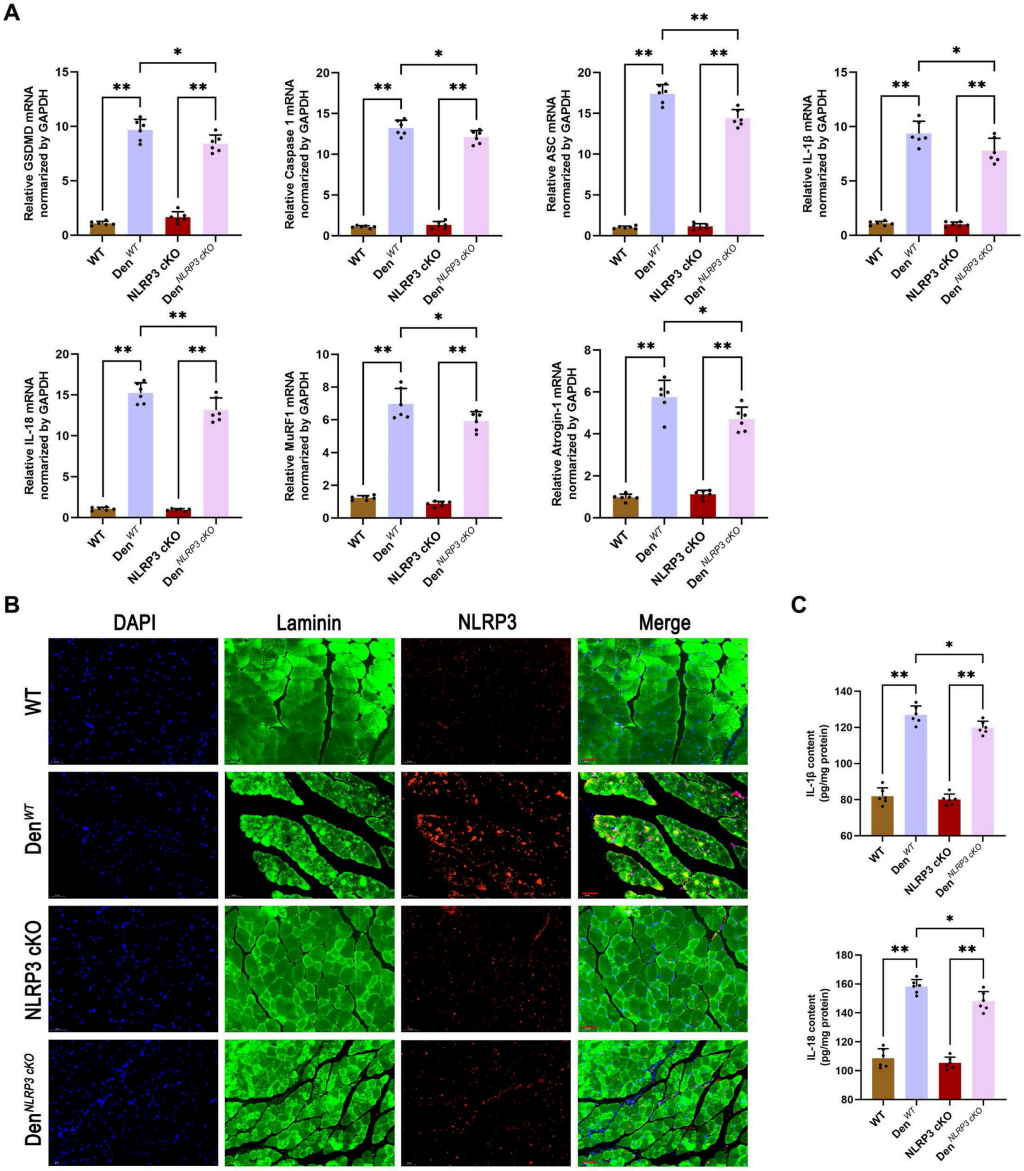
** NLRP3 cKO attenuates muscle atrophy and pyroptosis after denervation. (A)** Quantitative PCR analysis of mRNA levels of NLRP3, GSDMD, Caspase 1, ASC, IL-1β, IL-18, MuRF1 and Atrogin-1 in each group.** (B)** Representative immunofluorescence staining images of NLRP3 in each group. Scale bar, 50 μm. **(C)** Quantitative ELISA analysis of IL-1β and IL-18 in each group. Data are expressed as mean ± SD. n = 6 per group. *p < 0.05, **p < 0.01, ns *p* > 0.05. WT, wild type. Den, denervation.

To further elucidate the mechanisms underlying these morphological changes, Western blotting and PCR were performed to measure the expression of NLRP3-related molecules and atrophy markers in denervated GAS muscles from WT and NLRP3 cKO mice. As expected, the expression of NLRP3-mediated pyroptosis-related molecules, including GSDMD, Caspase 1, ASC, IL-1β and IL-18, was obviously reduced in the NLRP3 cKO group (Figure 4G-H; Figure 5A). Notably, NLRP3 cKO abolished the activation of GSDMD and Caspase 1 as well as the increase in MuRF1 and Atrogin-1 expression induced by denervation (Figure 4G-H; Figure 5A). Immunofluorescence staining and ELISA revealed similar changes, as shown in Figure 5B-C. These data demonstrated that NLRP3-dependent pyroptosis aggravates muscle atrophy and critically mediates catabolic pathways in muscles after denervation.

### Effects of NLRP3 inflammasome on GAS muscle function after denervation

To investigate the *in vivo* effects of NLRP3 inflammasome on GAS muscle function, the force transducer was employed *in situ* before sacrifice (Figure S1A). As is shown in Figure S1B, the denervation resulted in force loss and over the denervated time, the mice exhibit an increase in force drop compared to sham group. However, NLRP3 cKO in skeletal muscle did not elicit observable changes in GAS muscle function (Figure S1C), which indicated that NLRP3 inflammasome is not effective at preventing force drop during denervation.

### NLRP3 inflammasome-mediated pyroptosis induced by NIA in C2C12 myotubes* in vitro*

**Figure 6:**
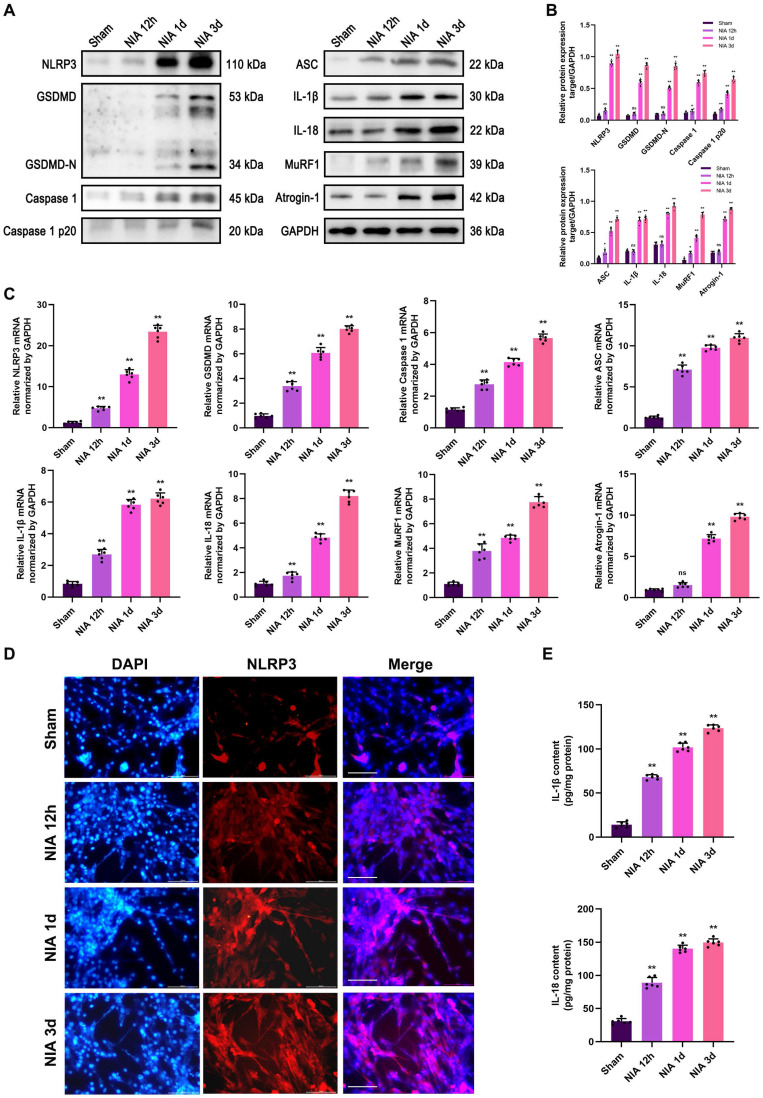
** NIA induces myocyte atrophy accompanied by pyroptotic molecules and UPS ligases upregulation in C2C12 myotubes. (A)** Western blot analysis of expression levels of NLRP3, GSDMD, GSDMD-N, Caspase 1, Caspase 1 p20, ASC, IL-1β, IL-18, MuRF1 and Atrogin-1 in C2C12 myotubes treated with NIA for 0, 12 h, 1 d and 3 d. **(B)** Quantification of NLRP3, GSDMD, GSDMD-N, Caspase 1, Caspase 1 p20, ASC, IL-1β, IL-18, MuRF1 and Atrogin-1. **(C)** Quantitative PCR analysis of mRNA levels of NLRP3, GSDMD, Caspase 1, ASC, IL-1β, IL-18, MuRF1 and Atrogin-1 in C2C12 myotubes treated with NIA for 0, 12 h, 1 d and 3 d. **(D)** Representative immunofluorescence staining images of NLRP3 in C2C12 myotubes treated with NIA for 0, 12 h, 1 d and 3 d. Scale bar, 50 μm. **(E)** Quantitative ELISA analysis of IL-1β and IL-18 in the supernatant of C2C12 myotubes after NIA treatment for 0, 12 h, 1 d and 3 d. Data are expressed as mean ± SD. n = 6 per group. *p < 0.05, **p < 0.01, ns *p* > 0.05 vs Sham group. NIA, NLRP3 inflammasome activator.

To verify the exact role of the NLRP3 inflammasome in myocyte atrophy, we exploited an *in vitro* model to determine whether the NLRP3 inflammasome exerts similar effects on skeletal myotubes. Here, we observed progressive upregulation of the expression of pyroptosis- and UPS-related molecules within 3 d in NIA-treated C2C12 myotubes (Figure 6A-C). Additionally, immunofluorescence staining of NLRP3 revealed that NLRP3 expression showed a trend, with an increase in expression that was extremely significant at 3 d (Figure 6D). Futhermore, NIA caused the secretion of massive amounts of IL-1β and IL-18 from C2C12 myotubes into the medium (Figure 6E). The above results suggested that the expression of UPS-related molecules may be triggered by NLRP3 inflammasome activation *in vitro*.

### Knockdown of NLRP3 ameliorated C2C12 myocyte atrophy induced by NIA

**Figure 7:**
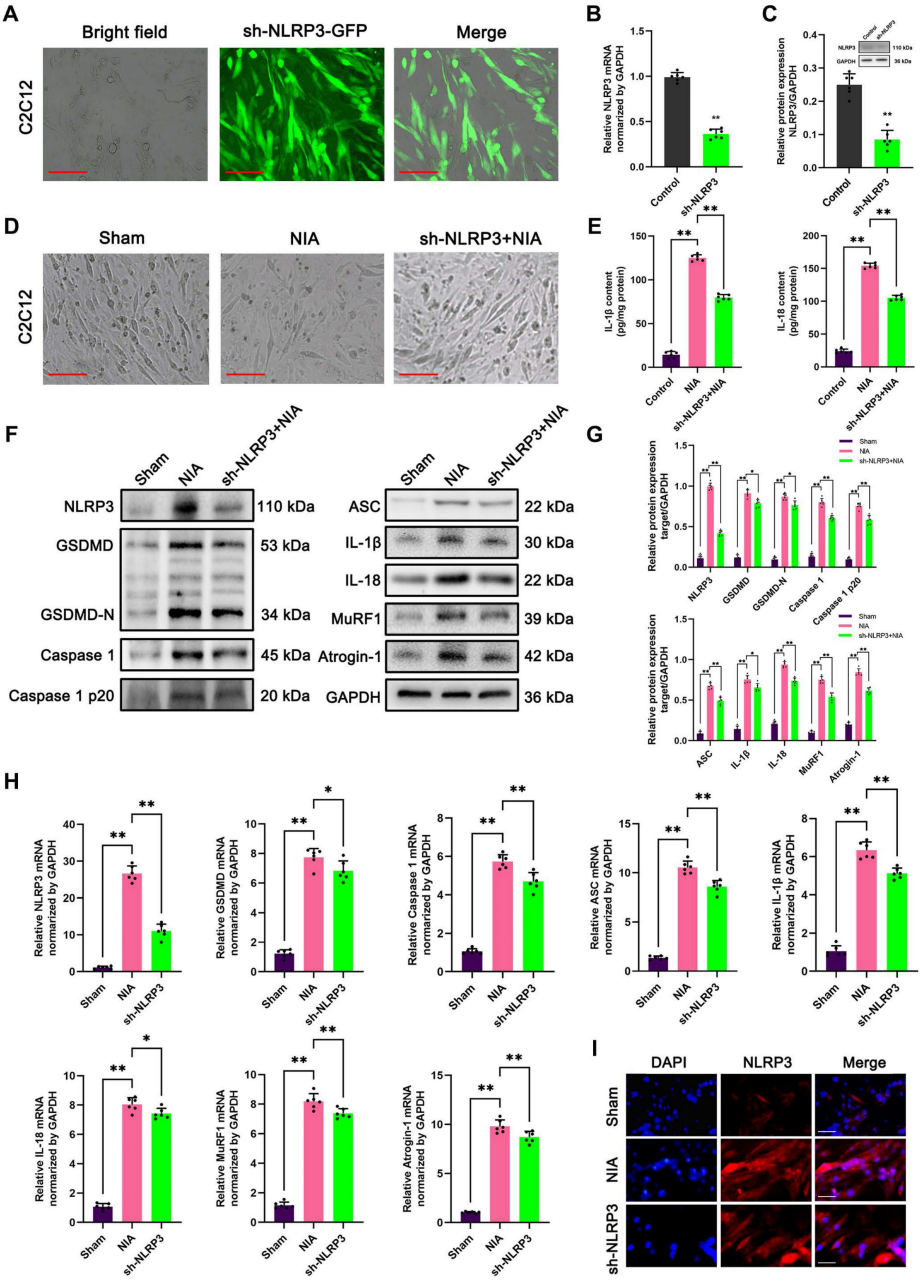
** Knockdown of NLRP3 ameliorates NIA-induced myocyte atrophy and pyroptosis in C2C12 myotubes. (A)** The transfection of sh-NLRP3 *in vitro*. Scale bar, 50 μm. **(B)** Quantitative PCR analysis confirmed the mRNA level of NLRP3 knockdown in C2C12 myotubes. **(C)** Western blot analysis confirmed the expression level of NLRP3 knockdown in C2C12 myotubes. **(D)** Bright field photomicrographs of C2C12 myotubes in each group. Scale bar, 50 μm. **(E)** Quantitative ELISA analysis of IL-1β and IL-18 in the supernatant of C2C12 myotubes in each group. **(F)** Western blot analysis of expression levels of NLRP3, GSDMD, GSDMD-N, Caspase 1, Caspase 1 p20, ASC, IL-1β, IL-18, MuRF1 and Atrogin-1 in each group. **(G)** Quantification of NLRP3, GSDMD, GSDMD-N, Caspase 1, Caspase 1 p20, ASC, IL-1β, IL-18, MuRF1 and Atrogin-1. **(H)** Quantitative PCR analysis of mRNA levels of NLRP3, GSDMD, Caspase 1, ASC, IL-1β, IL-18, MuRF1 and Atrogin-1 in each group. **(I)** Representative immunofluorescence staining images of NLRP3 in each group. Scale bar, 100 μm. Data are expressed as mean ± SD. n = 6 per group. *p < 0.05, **p < 0.01, ns *p* > 0.05 vs Sham group. NIA, NLRP3 inflammasome activator.

To further evaluate the involvement of the NLRP3 inflammasome in myocyte atrophy induced by NIA, we knocked down NLRP3 in C2C12 myotubes and asked whether myocyte atrophy is alleviated in the absence of NLRP3. Immunofluorescence staining combined with bright field microscopy revealed that the transfection efficiency of LV expressing sh-NLRP3 in the myotubes was high (Figure 7A). In addition, PCR and Western blotting confirmed that NLRP3 expression was markedly reduced in C2C12 myotubes (Figure 7B-C). Furthermore, bright field microscopy clearly showed that myocyte atrophy was significantly aggravated by NIA treatment and was remarkedly reversed after NLRP3 knockdown (Figure 7D). Consistently, quantitative analysis revealed that the expression of pyroptosis pathway-associated molecules and E3 ubiquitin ligases (Atrogin-1 and MuRF1) was notably inhibited in NLRP3 knockdown cells (Figure 7E-I). These data demonstrated that the anti-atrophy effect of NLRP3 knockdown in C2C12 myotubes might result from a reduction in IL-1β secretion and suppression of UPS activation in C2C12 myotubes, which is consistent with previous reports of the role of NLRP3/IL-1β function in atrophy 14.

### NLRP3 inflammasome activation inhibited apoptosis in denervated GAS muscles and NIA-treated C2C12 myotubes

**Figure 8:**
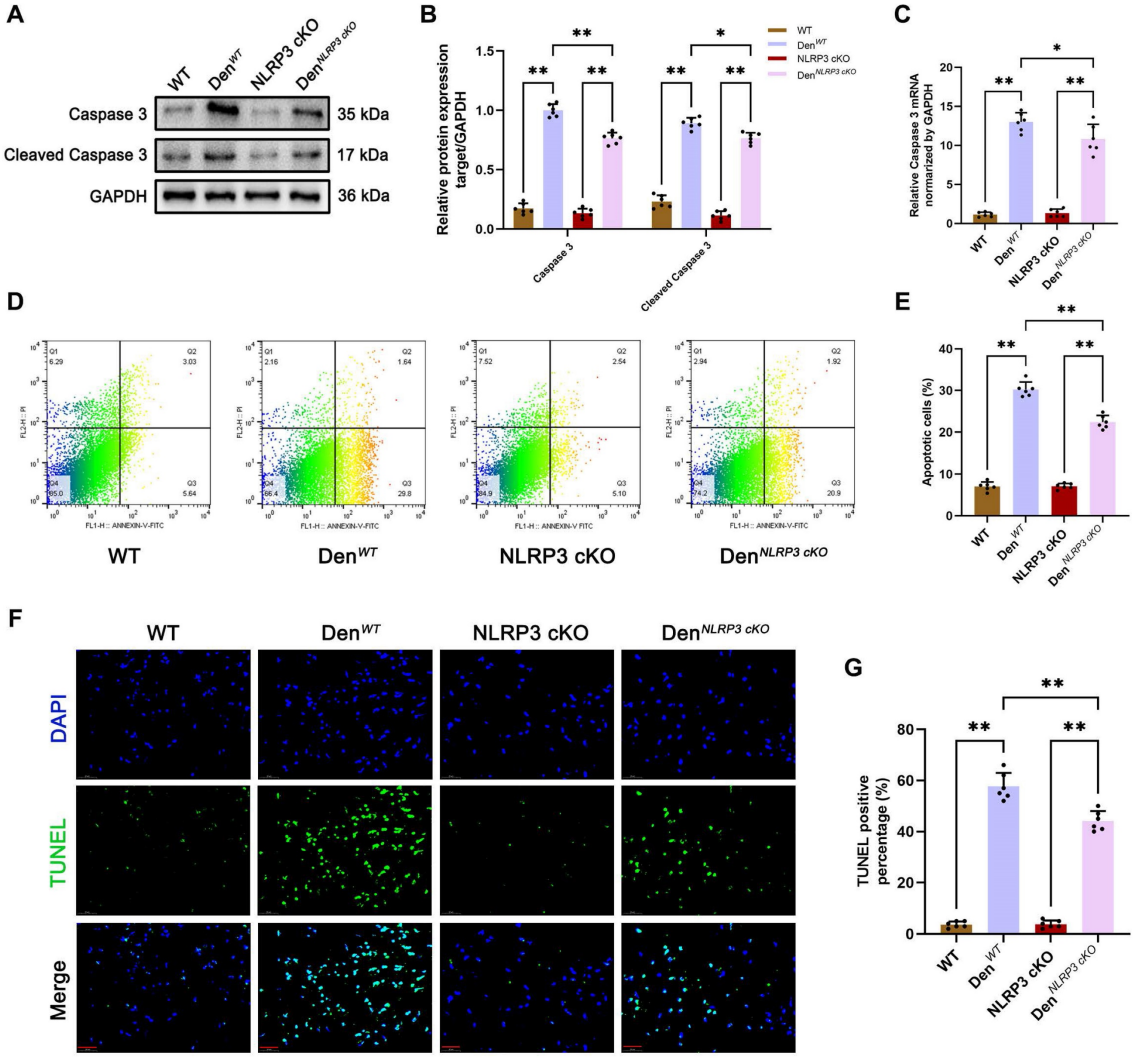
** NLRP3 cKO alleviates apoptosis after denervation. (A)** Western blot analysis of expression levels of caspase 3 and cleaved caspase 3 in each group. **(B)** Quantification of caspase 3 and cleaved caspase 3.** (C)** Quantitative PCR analysis of mRNA levels of caspase 3 in each group. **(D)** Flow cytometry analysis of apoptosis in each group. **(E)** The ratio of apoptotic cells in each group. **(F)** Representative photomicrographs of TUNEL staining. Scale bar, 50 μm. **(G)** Percentage of TUNEL-positive cells. Data are expressed as mean ± SD. n = 6 per group. *p < 0.05, **p < 0.01, ns *p* > 0.05. WT, wild type. Den, denervation.

Given that various studies have reported that NLRP3 inflammasome activation is closely associated with cell apoptosis 20, 21, we further examined the effects of NLRP3 on apoptosis in muscle tissues after denervation *in vivo* and *in vitro.* We found that the levels of the classical apoptosis-related molecule caspase 3 and its cleaved form were increased in WT and NLRP3 cKO mice after denervation, as determined by Western blotting and PCR (Figure 8A-C). In contrast, the levels of these molecules were dramatically reduced in NLRP3 cKO mice compared to WT mice after denervation (Figure 8A-C). As showed in Figure 8D-E, flow cytometry showed that cells underwent apoptosis after denervation (Figure 8D-E). However, the rate of cell apoptosis was decreased in denervated GAS muscle tissues from NLRP3 cKO mice compared with those from WT mice (Figure 8D-E). Consistent with the flow cytometry results, TUNEL staining revealed that cell apoptosis in GAS muscles was attenuated by NLRP3 cKO mice after denervation (Figure 8F-G).

**Figure 9:**
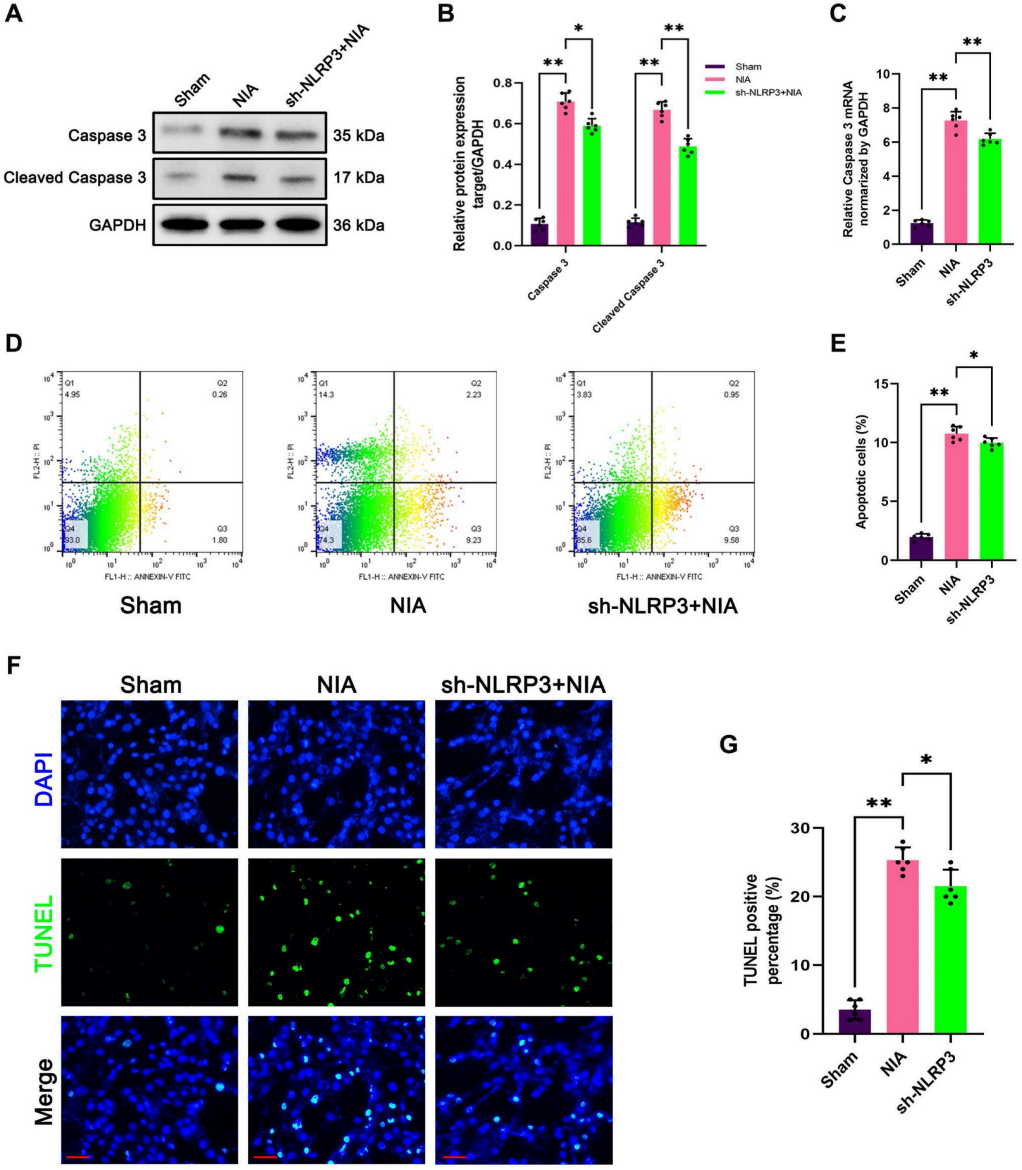
** Knockdown of NLRP3 mitigates NIA-induced apoptosis in C2C12 myotubes. (A)** Western blot analysis of expression levels of of caspase 3 and cleaved caspase 3 in each group. **(B)** Quantification of caspase 3 and cleaved caspase 3. **(C)** Quantitative PCR analysis of mRNA levels of caspase 3 in each group. **(D)** Flow cytometry analysis of apoptosis in each group. **(E)** The ratio of apoptotic cells in each group. **(F)** Representative photomicrographs of TUNEL staining. Scale bar, 50 μm. **(G)** Percentage of TUNEL-positive cells. Data are expressed as mean ± SD. n = 6 per group. *p < 0.05, **p < 0.01, ns *p* > 0.05. NIA, NLRP3 inflammasome activator.

Similarly, upregulation of caspase 3 and cleaved caspase 3 expression has been implicated in the induction of myocyte atrophy by NIA (Figure 9A-C). As expected, knockdown of NLRP3 by sh-NLRP3 decreased the levels of caspase 3 and cleaved caspase 3 in NIA-treated C2C12 myotubes (Figure 9A-C). Moreover, the flow cytometry results showed that NIA-mediated apoptosis was significantly alleviated after NLRP3 knockdown in C2C12 myotubes (Figure 9D-E). Furthermore, the number of TUNEL-positive nuclei was markedly reduced in NIA-treated myotubes (Figure 9F-G).

In summary, these findings revealed a close connection between NLRP3 inflammasome activation and apoptosis, which may contribute to muscle atrophy *in vivo* and *in vitro*.

## Discussion

Disability resulting from peripheral nerve injury, such as skeletal muscle atrophy, has attracted increasing interest, particularly among those who are prone to peripheral nerve injury 22. The molecular mechanisms responsible for the changes that occur after peripheral nerve injury remain largely unknown. Several studies have recently examined the vital role of the NLRP3 inflammasome in muscle atrophy 23, 24, but fewer studies have explored its role in denervation. Thus, we aimed to carefully analyse whether the NLRP3 inflammasome influences muscle mass to provide novel insight into the mechanism of denervation.

According to existing evidence, in rats, the atrophy process can be divided into four different stages that occur within a period of 28 d after denervation 19, indicating the occurrence of a pathological cascade. Of equal importance are our findings that reductions in muscle mass and fibre diameter occurred beginning at 7 d and were followed by rapid muscle weight loss over the following two weeks after sciatic section in mice. This result is somewhat inconsistent with previously published work demonstrating rapid weight loss during the first two weeks after denervation 25. Our data might instead suggest that the orchestrated muscular events and complex interactions that occur two weeks after denervation are of greater importance, and the morphological changes in atrophied GAS muscles presented in this study confirm this hypothesis. Consequently, we focused on elucidating the mechanism underlying the later stage (14~28 d) after denervation-induced muscle atrophy.

In recent years, DEG analysis has proven to be a valuable method for evaluating pathogenesis. although many studies have examined DEGs in atrophic muscles 26, 27, detailed information on the mechanism underlying denervation is lacking. Our analyses of high-throughput data from denervated GAS muscles revealed that NLRP3 inflammasome complex assembly is positively regulate during the later stage of denervation. According to previous reports, assembly of the NLRP3 inflammasome triggers proteolytic cleavage of dormant pro-caspase-1 into active caspase-1, which converts the cytokine precursors pro-IL-1β and pro-IL-18 into mature and biologically active IL-1β and IL-18, respectively 28. On the one hand, mature IL-1β is a potent proinflammatory mediator in many pathologic processes; on the other hand, active caspase 1 can induce a new form of cell death called pyroptosis 29. Based on these findings, we speculate that NLRP3 inflammasome activation induced by muscular denervation plays a crucial role in muscle atrophy partly through inflammation and pyroptosis.

To our knowledge, Nora et al. performed immunostaining and reporter gene assays using atrophic muscles from WT and NLRP3 knockout mice with septicaemia for the first time and reported that NLRP3 inflammasome activation is one of the mechanisms leading to muscle atrophy 12. Thereafter, emerging studies have demonstrated that NLRP3 inflammasome activation is a major cause of inflammation and pyroptosis, leading to cell death and dysfunction of myocyte and culminating in muscle atrophy 30, 31. Furthermore, blockade of the NLRP3/GSDMD pathway has been shown to attenuate muscle atrophy by decreasing the expression of the UPS-related ligases MuRF1 and Atrogin-1 23. Thereinto, the UPS is considered to be the most prominent process related to muscle atrophy 32. As expected, our data clearly revealed that the NLRP3 inflammasome was significantly activated and that the levels of NLRP3-mediated pyroptosis-related molecules in the GAS muscle progressively increased after denervation, resulting in elevated expression of MuRF1 and Atrogin-1. *In vitro* NIA-induced myocyte atrophy resulted in similar changes as those observed *in vivo*. Specifically, inhibiton of pyroptosis via knockdown or knockout of NLRP3 ameliorated muscle atrophy in mice and myotubes, respectively. This finding reveals that NLRP3 inflammasome activation induced by denervation contributes to pyroptotic cell death and protein degradation, resulting in muscle atrophy.

Another intriguing finding of our study is that NLRP3 inflammasome activation affects apoptosis in the GAS muscle following denervation. Apoptosis is a form of programmed cell death that plays an important role in a variety of biological processes, including tissue turnover, immunological defence and embryonic development 33. Thereinto, a cascade that culminates in caspase 3 activation mediates apoptosis and is responsible for cell death. Cells that undergo apoptosis are quickly engulfed for degradation. In particular, accumulating studies have demonstrated the involvement of apoptosis in the loss of postmitotic skeletal muscle after denervation 34. Herein, we found that the expression of caspase 3 and cleaved caspase 3 in denervated GAS muscles was down-regulated by loss of NLRP3 *in vivo* and *in vitro*. Furthermore, flow cytometry and TUNEL staining confirmed that cells underwent apoptosis, suggesting a crucial role for the NLRP3 inflammasome in denervation-induced apoptosis.

Although the NLRP3 inflammasome-mediated pyroptosis might account for the atrophied effects seen here, mechanistic evidence on series connection among pyroptosis, proteolysis and apoptosis remain scarce. Previously, HMGB1-induced pyroptosis was reported to contribute in proteolysis development and sarcopenia progression, in which the sterile inflammation resulting from cytokines such as IL-1β and IL-6 act as intermediate role 13. Also, Nora et al. identified that MuRF1 protein expression in gastrocnemius/plantaris kept unchanged by inhibition of IL-1β in NLRP3 KO mice of sepsis 12. These observations coincided with improved myopathies in our study indicative for a close pyroptosis-proteolysis relationship. Furthermore, as recently reported by Li et al., NLRP3 deficiency significantly alleviates iohexol-induced renal apoptosis 20, potentially via the Caspase 1/mitochondria pathway 35. Consistent with our study, moreover, Ning Li et al. confirmed that the expression of C-Caspase3 was positively regulated by NLRP3 inflammasome activation, indicating that Caspase8/3 induced apoptosis can be regarded as an intrinsic part of inflammasome function 21. Both of above seems emphasize the crucial role of NLRP3 inflammasome in pyroptosis-apoptosis interacting. It follows that although comprehensive data in present study provides an updated insight of NLRP3 inflammasome on anti-apoptosis efficacy in denervated muscles, the specific molecular mechanism remains unclear yet. Hence, further experiments need to be investigated and specific genetical mice might be more persuasive.

In addition, the functional investigation in this study demonstrated that blocking NLRP3 inflammasome is not effective at reducing force loss of GAS muscles. To our knowledge, it is plausible that the inflammasomes are likely involved in the inflammatory microenvironment and programmed death, whereas, have little influence on membrane excitability, depolarization and potential overshoot 28, 36. Similarly, via targeting inflammatory signalling pathways, several studies have failed to prevent atrophy process and rescue muscle function at the same time 37-39. It should be feasible to combine inhibiting NLRP3 inflammasome activation with some other approaches, as correction, even with gene therapy, is expected to be incomplete. Our results are perhaps a better representation of the therapeutic strategy in the pathological changes of pyroptosis, proteolysis and apoptosis within a long denervation period. For denervated muscle atrophy, hereby, onging efforts are aimed at preventing both muscle mass loss and force reduction.

## Conclusions

**Figure 10:**
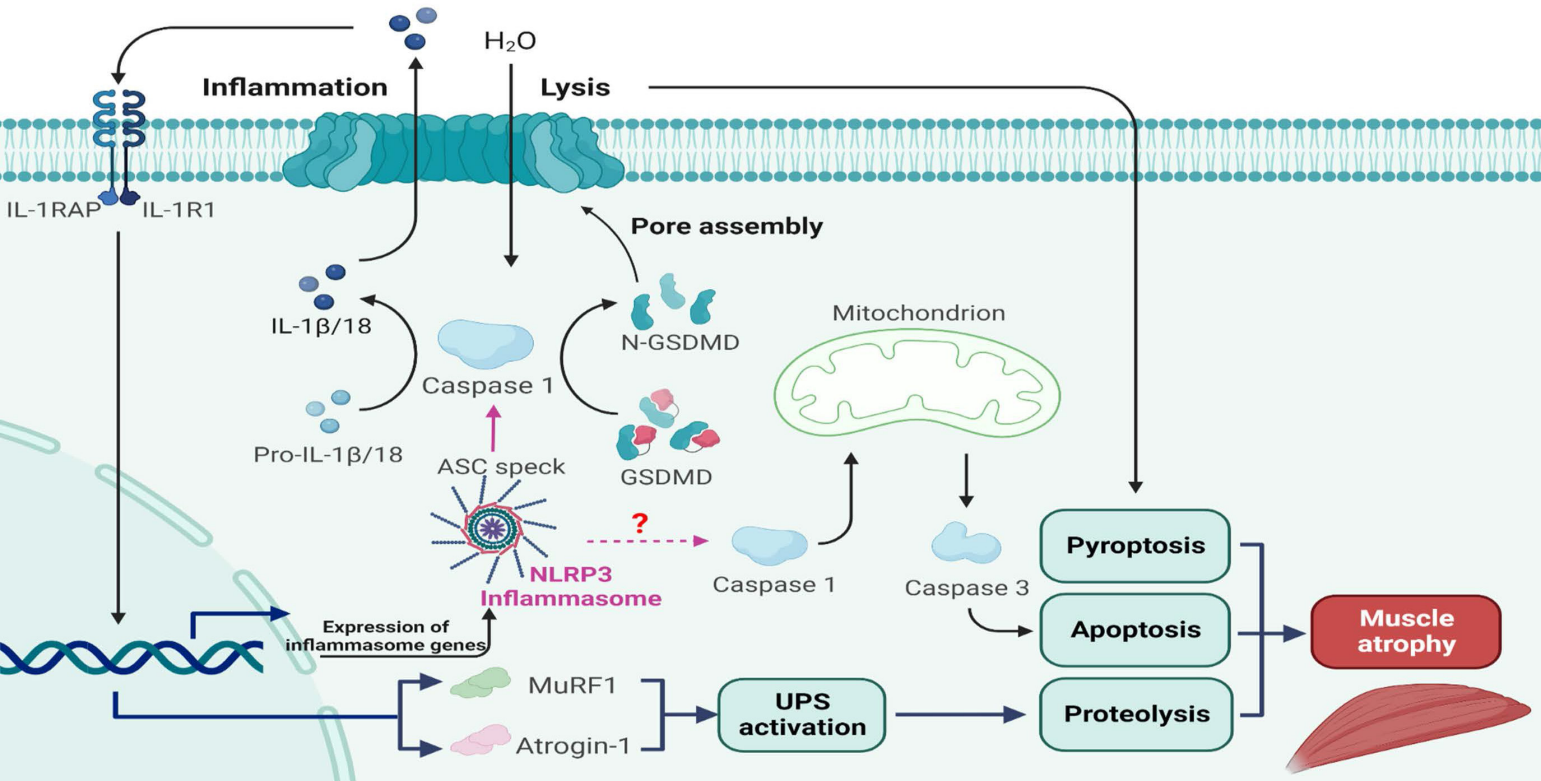
A schematic diagram of the mechanism by which NLRP3 inflammasome activation aggravates denervated muscle atrophy via pyroptosis, apoptosis and proteolysis.

Taken together, the results of the present study for the first time show that the NLRP3 inflammasome is activated following denervation, and that a progressive increase in NLRP3 inflammasome activity aggravates muscle atrophy over time. Mechanistically, NLRP3 inflammasome activation leads to severe muscle alterations related to inflammation, pyroptosis, proteolysis and apoptosis after denervation (Figure 10). Analysis of C2C12 myotube atrophy induced by NIA further validated these results. Moreover, muscle-specific NLRP3 cKO and NLRP3 knockdown reversed the atrophic myopathy *in vivo* and *in vitro*, respectively. Therefore, targeting NLRP3 inflammasome activation is a potential therapeutic strategy for ameliorating muscle atrophy post denervation.

## Supplementary Material

Supplementary figure.

## Figures and Tables

**Table 1:** Real-time PCR primers used in this study

Gene	Primers, 5'-3'	
	Forward	Reverse
*NLRP3*	ATTACCCGCCCGAGAAAGG	TCGCAGCAAAGATCCACACAG
*ASC*	CTCCTCAGATGAAGGGCTTG	CCCTGGGAAAGTGGGTTATT
*GSDMD*	TGCGTGTGACTCAGAAGACC	ATAAAGCTCCAGGCAGCGTA
*Caspase 1*	ACAAGGCACGGGACCTATG	TCCCAGTCAGTCCTGGAAATG
*IL-1β*	GCAACTGTTCCTGAACTCAACT	ATCTTTTGGGGTCCGTCAACT
*IL-18*	GACTCTTGCGTCAACTTCAAGG	CAGGCTGTCTTTTGTCAACGA
*MuRF1*	GTGTGAGGTGCCTACTTGCTC	GCTCAGTCTTCTGTCCTTGGA
*Atrogin-1*	CAGCTTCGTGAGCGACCTC	GGCAGTCGAGAAGTCCAGTC

## Abbreviations

NLRP3: Nod-like receptor protein 3
cKO: conditional knock out
GAS: gastrocnemius
WB: western blot
PCR: polymerase chain reaction
ELISA: enzyme linked immunosorbent assay
TUNEL: TdT-mediated dUTP Nick-End Labeling
NIA: NLRP3 inflammation activator
UPS: ubiquitin proteasome system
GSDMD: gasdermin D
IL-1β: interleukin 1 beta
IL-18: interleukin 18
ATCC: American Type Culture Collection
LV: lentiviral
HE: haematoxylin-eosin
ASC: apoptosis-associated speck-like protein containing CARD
GAPDH: glyceraldehyde 3-phosphate dehydrogenase
DAPI: 4,6-diamidino-2-phenylindole
FITC: fluorescein isothiocyanate
PI: propidium iodide
DEGs: differential expressed genes
GO: gene ontology
BP: biological process
WT: wild-type
