# Supplementary material for: Ablation of NLRP3 inflammasome attenuates muscle atrophy via inhibiting pyroptosis, proteolysis and apoptosis following denervation
Source: Theranostics. 2023 Jan 1;13(1):374–90. doi: 10.7150/thno.74831 (PMC9800723; doi:10.7150/thno.74831)
Supplement: Supplementary file 1 — Supplementary figure. [file thnov13p0374s1.pdf]

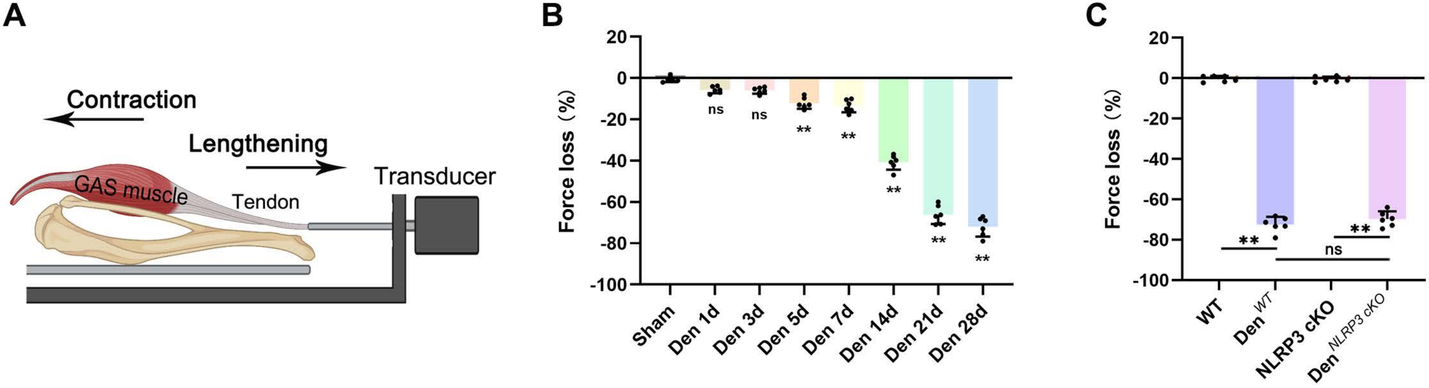
A
Contraction
Lengthening
GAS muscle
Tendon
Transducer
B
Force loss (%)
20
0
-20
-40
-60
-80
-100
ns
ns
**
**
**
**
**
Sham
Den 1d
Den 3d
Den 5d
Den 7d
Den 14d
Den 21d
Den 28d
C
Force loss (%)
20
0
-20
-40
-60
-80
-100
**
**
ns
WT
Den WT
NLRP3 cKO
Den NLRP3 cKO

Figure S1. Effects of NLRP3 inflammasome on GAS muscle function after denervation. (A) The visualized diagram for force measurement in mice after denervation. (B) The force loss of the GAS muscles at indicated time points after denervation. (C) The amount of force loss in GAS muscles after denervation in each group. Data are expressed as mean ± SD. n = 6 per group. *$p < 0.05$, **$p < 0.01$, ns $p > 0.05$.
